# Feasibility of stepwise baricitinib tapering after complete response in alopecia areata: A real-world observational cohort study

**DOI:** 10.1016/j.jdin.2026.07.005

**Published:** 2026-07-22

**Authors:** Hye Rin Seo, Do-Young Kim

**Affiliations:** Department of Dermatology and Cutaneous Biology Research Institute, Yonsei University College of Medicine, Seoul, Republic of Korea

**Keywords:** alopecia areata, baricitinib, dose reduction, epidemiology, Janus kinase inhibitor, real-world study

*To the Editor:* Alopecia areata (AA) is a relapsing immune-mediated disorder, and management after complete regrowth on Janus kinase inhibitors remains uncertain. Although maintenance therapy is often recommended, real-world data on baricitinib tapering after complete response (CR) are limited.^1^^,^^2^ We evaluated stepwise baricitinib tapering in patients with AA who achieved CR on baricitinib 4 mg daily.

In this institutional review board–approved single-center observational study at Severance Hospital (Seoul, Korea; No. 4-2025-0607), we included 66 patients who initiated tapering between December 2023 and February 2026. Eligible patients had maintained dermatologist-assessed clinical CR (Severity of Alopecia Tool [SALT] 0 or near-zero with minimal inactive areas) on baricitinib 4 mg daily for at least 2 months. Follow-up continued through April 30, 2026. Tapering used a predefined sequence with 6 to 8–week reassessments: 4 mg daily, alternating 4-mg/2-mg dosing, 2 mg daily, 2 mg 5 times weekly, 2 mg every other day, and discontinuation. Further reduction was considered when sustained CR persisted without new activity. Minor recurrence (SALT <5) was managed with local therapy/step retention or dose escalation; major recurrence (SALT ≥5) prompted 4-mg re-escalation. Details are in the Supplementary Methods, available via Mendeley at https://data.mendeley.com/datasets/x6fs7g3jvh/1.

The primary outcome was tapering failure, defined as recurrence prompting 4-mg re-escalation. Among 66 patients (Table I), median age at onset was 29.5 years (interquartile range [IQR], 13.2-39.2), baseline SALT score was 25.0 (IQR, 10.0-71.2), and median current episode duration was 11.3 months (IQR, 4.6-25.3). Median time from baricitinib initiation to tapering was 7.1 months (IQR, 5.3-10.4). Tapering failure occurred in 19/66 (28.8%) patients (median time to failure, 3.8 months [IQR, 2.5-6.2]); 47/66 (71.2%) avoided re-escalation to 4 mg, including 15/22 with baseline SALT >50 (Supplementary Table I, available via Mendeley at https://data.mendeley.com/datasets/7z7srk959f/1). Failure tended to be more frequent (31.8%) in severe disease (SALT >50). Of the 19 failures, 16 (84.2%) were minor recurrences (SALT <5) and 3 major recurrences occurred only in refractory, rapidly progressive alopecia totalis. Overall, 46/66 patients (69.7%) maintained response at 2 mg or lower, including 12 (18.2%) off-drug successes (median follow-up after discontinuation, 4.0 months [IQR, 2.7-5.0]). Exploratory analyses suggested differences by disease course (*P* = .015; Table I) and current episode duration (*P* = .017; Fig 1).

**Table I:** Baseline characteristics according to tapering outcome

Variable	Total (*N* = 66)	Tapering failure (*n* = 19)	Tapering success (*n* = 47)		*P* value
			On-drug success (*n* = 35)	Off-drug success (*n* = 12)	
Demographics					
Age at onset, y	29.5 (13.2-39.2)	29.0 (12.0-40.0)	29.0 (18.5-35.5)	36.5 (12.0-51.5)	.637
Female sex	42 (63.6)	14 (73.7)	22 (62.9)	6 (50.0)	.399
BMI, kg/m^2^	22.4 (19.8-24.2)	22.6 (20.0-24.0)	21.9 (19.6-24.6)	23.1 (21.0-24.2)	.818
Disease characteristics					
Alopecia subtype					.338
Patch-type AA	30 (45.5)	5 (26.3)	19 (54.3)	6 (50.0)	
Alopecia totalis	14 (21.2)	7 (36.8)	4 (11.4)	3 (25.0)	
Alopecia universalis	10 (15.2)	3 (15.8)	6 (17.1)	1 (8.3)	
Ophiasis	12 (18.2)	4 (21.1)	6 (17.1)	2 (16.7)	
Disease course					.015∗
Rapid progression	12 (18.2)	6 (31.6)	2 (5.7)	4 (33.3)	
Gradual/chronic progression	54 (81.8)	13 (68.4)	33 (94.3)	8 (66.7)	
Total AA duration, mo	39.6 (10.8-122.2)	25.3 (14.2-49.3)	70.9 (12.8-143.6)	25.9 (4.7-103.3)	.462
Current episode duration, mo	11.3 (4.6-25.3)	18.5 (6.9-35.2)	13.2 (3.4-54.1)	5.0 (2.4-11.6)	.017∗
Baseline SALT score	25.0 (10.0-71.2)	36.5 (10.0-95.0)	25.0 (10.8-60.8)	19.0 (12.0-50.2)	.894
Treatment characteristics					
Time from baricitinib initiation to tapering, mo	7.1 (5.3-10.4)	8.4 (5.8-12.6)	6.7 (5.4-10.9)	5.4 (4.8-6.7)	.167
Follow-up duration after taper initiation, mo	9.5 (6.4-15.6)	9.3 (5.6-15.8)	9.4 (6.3-15.0)	10.8 (8.4-19.9)	.371

Data are presented as median (IQR) or *n* (%), unless otherwise indicated. *P* values compare the 3 tapering outcome groups. Continuous variables were compared using the Kruskal-Wallis test. Categorical variables were compared using the Fisher exact test or Fisher-Freeman-Halton test, as appropriate. *P* values were exploratory and were not adjusted for multiple comparisons. Baseline SALT score was available for 64 patients. Rapid progression was defined as progression to alopecia totalis within 3 months of AA onset. On-drug success was defined as avoidance of re-escalation to 4 mg daily while continuing baricitinib at a reduced dose at last follow-up; off-drug success was defined as baricitinib discontinuation without re-escalation at the last follow-up.

*AA*, Alopecia areata; *BMI*, body mass index; *IQR*, interquartile range; *SALT*, Severity of Alopecia Tool.

∗ *P* < .05.

**Fig 1:**
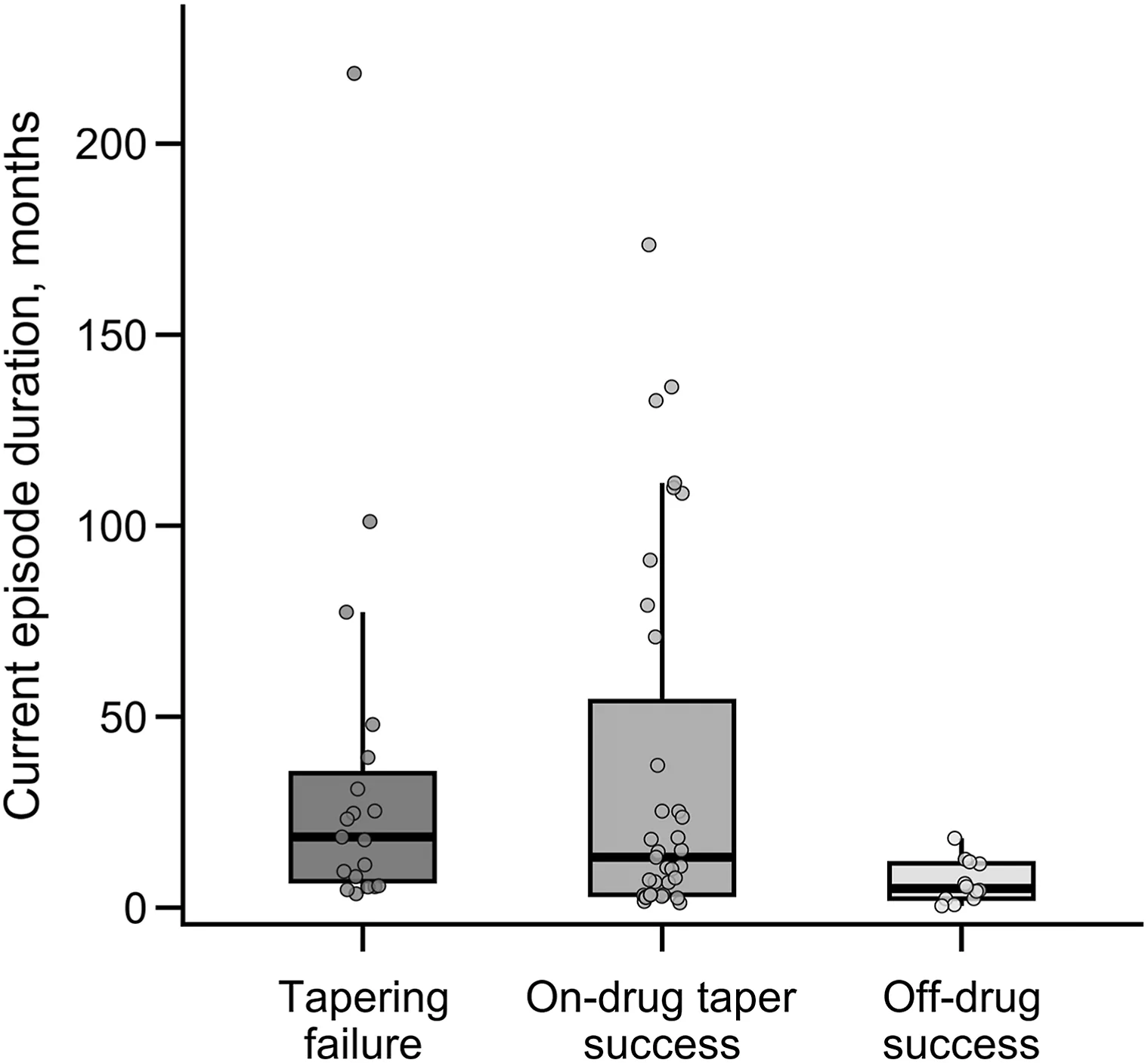
Alopecia areata: current episode duration according to tapering outcome after clinically complete response on baricitinib 4 mg. *Boxes* indicate the median and interquartile range, whiskers extend to values within 1.5 times the interquartile range, and *dots* indicate individual patients. Groups were compared using the Kruskal-Wallis test.

These findings suggest that baricitinib tapering after CR appears feasible in practice. Unlike the BRAVE-AA trials, which evaluated downtitration or withdrawal after achieving SALT ≤20, our strategy prioritized disease stability by initiating tapering only after 2 months of sustained CR.^3^^,^^4^ This more conservative threshold and strategy likely explain why most failures in our cohort were early and minor, supporting the feasibility of stepwise reduction. Shorter current episode duration may be relevant to tapering success, although this exploratory association requires validation. Korea-specific cost analysis showed a median 34.3% (IQR, 21.8% to 49.0%) reduction in patient-borne baricitinib expense (Supplementary Table II, available via Mendeley at https://data.mendeley.com/datasets/rdnx5h4jxk/1). Notably, no new safety signals emerged during tapering (Supplementary Table III, available via Mendeley at https://data.mendeley.com/datasets/zykgn9rzcs/1). Limitations include the single-center design, limited follow-up, unadjusted exploratory analyses, Korea-specific cost estimates, and lower baseline SALT scores than BRAVE-AA participants; however, prior systemic immunosuppression may have underestimated baseline severity. Nevertheless, these real-world data address a practical gap regarding post-CR management and suggest that stepwise tapering may minimize long-term treatment burden while maintaining clinical response.

## Conflicts of interest

None disclosed.

## References

[1] Harries M.J., Ahmed A., Ascott A. (2026). British Association of Dermatologists living guideline for managing people with alopecia areata 2025. Br J Dermatol.

[2] Kushnir-Grinbaum D., Bokhari L., Frewen J. (2025). Systemic treatment of moderate to severe alopecia areata in adults: updated Australian expert consensus statement. Australas J Dermatol.

[3] King B., Ko J., Kwon O. (2024). Baricitinib withdrawal and retreatment in patients with severe alopecia areata: the BRAVE-AA1 randomized clinical trial. JAMA Dermatol.

[4] King B., Ohyama M., Senna M. (2025). Outcomes of down-titration in patients with severe scalp alopecia areata initially treated with baricitinib 4-mg: week 152 data from BRAVE-AA2. J Am Acad Dermatol.

